# Function and Diversification of MADS-Box Genes in Rice

**DOI:** 10.1100/tsw.2006.320

**Published:** 2006-07-06

**Authors:** Takahiro Yamaguchi, Hiro-Yuki Hirano

**Affiliations:** ^1^ National Institute for Basic Biology, Okazaki, Aichi 444-8585, Japan; ^2^ Graduate School of Science, University of Tokyo, Hongo, Tokyo 113-8654, Japan

**Keywords:** Oryza sativa (rice), MADS-box gene, OsMADS, flower development, inflorescence development, DROOPING LEAF, ABC model, Poaceae (grass)

## Abstract

MADS-box genes play critical roles in a number of developmental processes in flowering plants, such as specification of floral organ identity, control of flowering time, and regulation of fruit development. Because of their crucial functions in flower development, diversification of the MADS-box gene family has been suggested to be a major factor responsible for floral diversity during radiation of the flowering plants. Inflorescences and flowers in the grass species have unique structures that are distinct from those in eudicots. Thus, it is plausible that the diversification of the function of MADS-box genes may have been a key driving force in the morphological divergence of the flowers and inflorescences in the grasses. Indeed, recent progress in genetic studies has shown that MADS-box genes function in flower development in *Oryza sativa* (rice), in support of the idea that functional diversification of the MADS-box genes was involved in evolution of the angiosperms. In this review, we summarize the functions of the major subfamilies of the MADS-box genes in rice and discuss their role in the development and evolution of rice flowers and inflorescences.

## INTRODUCTION

There are over 250,000 angiosperms extant today, and these plants have evolved a wide variety of flowers and inflorescences. Functional analyses by molecular genetic studies in model eudicots, such as *Arabidopsis thaliana*, have shown that transcription factors encoded by MADS-box genes are essential for the regulation of various aspects of flower development. It is of great interest to study MADS-box gene function in species distantly related to *Arabidopsis*, such as monocots, because the functional diversification of MADS-box genes has been proposed to be a major driving force behind floral diversity[1].

The monocots comprise about 50,000 species. A large number of MADS-box genes have been isolated in monocots and functional studies have been carried out. Most of these studies have focused mainly on the expression of genes homologous to the *Arabidopsis* genes that specify organ identity. Genetic approaches, however, are now more powerful for elucidating the actual function of the genes that regulate developmental processes. Indeed, much progress has been recently made in *Oryza sativa* (rice) and *Zea mays* (maize), two species of the grass family Poaceae, owing to the availability of genetic approaches. Rice has an additional advantage in developmental studies because the genetic transformation of rice is relatively easy and also facilitates the use of reverse genetic studies. In addition, rice is the only grass plant whose genome has been fully sequenced, and a number of rice MADS-box genes have been isolated experimentally or identified from the DNA database[2] (Fig. 1). Thus, functional studies using loss-of-function mutants or transgenic plants have revealed many novel aspects of MADS-box gene function (reviewed in[3,4]).

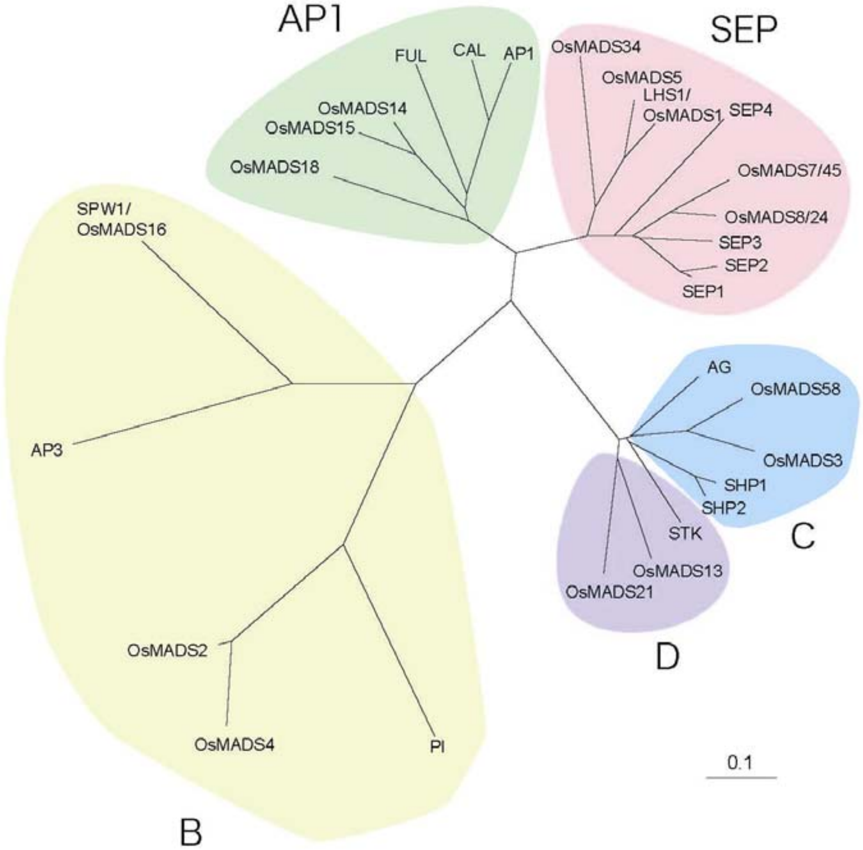

**FIGURE 1.** Phylogeny of the major classes of MADS-domain proteins from rice and *Arabidopsis.*

Grass species have flowers and inflorescences that are highly distinct from those of eudicots. Furthermore, the flowers and inflorescences of rice and maize have also morphologically diversified to some degree. Thus, it is plausible that comparative studies between grasses and eudicots, and between rice and maize, may reveal clues to the diversification of both MADS-box gene function and developmental pathways during evolution.

In this review, we summarize the results from studies on MADS-box gene functions in rice, focusing on the most recent progresses, and discuss the roles of MADS-box genes in the evolution of rice flowers and inflorescences.

## RICE SPIKELET AND FLORET

First, it is helpful to describe the structure of rice spikelets and florets, which have unique morphologies distinct from those of eudicot flowers. The spikelet is the structural unit of inflorescence and is composed of florets and glumes (rudimentary organs in rice). The rice spikelet is thought to have three florets[3,5], which are subtended by two tiny glumes (rudimentary glumes) (Fig. 2). The uppermost floret is bisexual and fertile, whereas the lower two florets are strongly reduced and sterile. The lower two florets develop only a reduced lemma, known as an "empty glume" (or sterile lemma). The uppermost floret consists of one lemma, one palea, two lodicules, six stamens, and one central pistil. The palea, lemma, and lodicule organs are unique to the grass family, but the palea and lemma are thought to correspond most closely to a prophyll and bract, respectively[6]. The lodicule is considered to be homologous to the petal in eudicots and is formed only at the lemma side. Although the stamen and carpel are highly conserved organs in angiosperms, the structure of the carpel of rice is diversified from those of eudicots.

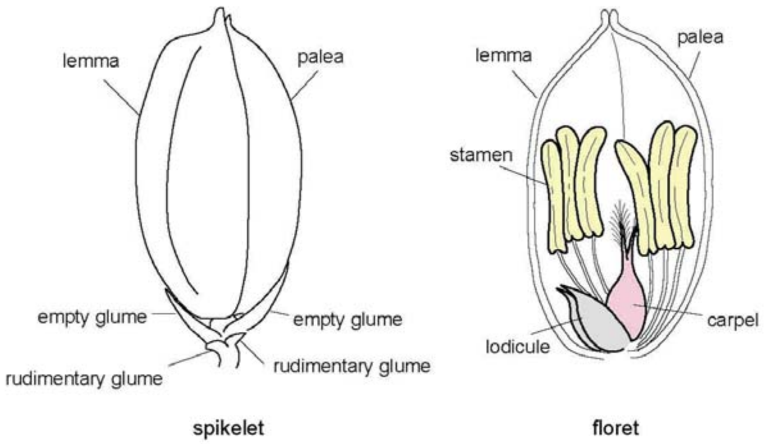

**FIGURE 2.** Morphology of the spikelet and floret in rice. The spikelet shows an outward form and the floret shows floral organs inside of the palea and lemma.

## *AP1*-LIKE GENES IN RICE

In the core eudicot *Arabidopsis*, the A-class MADS-box gene *APETALA1* (*AP1*) is required for establishment of the floral meristem and for specification of sepal and petal identity[7]. Specification of floral meristem identity in *Arabidopsis* is redundantly regulated by the *AP1* homologs *CAULIFLOWER* (*CAL*) and *FRUITFUL* (*FUL*)[8,9]. *FUL* is also involved in a distinct developmental process in fruit and inflorescence development[10]. In grass species, some *AP1*-like genes, such as *VRN1* of *Triticum monococcum* or *WAP1* of *T. aestevum*, are involved in the phase transition from vegetative to reproductive growth[11,12].

The rice genome contains at least three *AP1*-like MADS-box genes, *OsMADS14*, *OsMADS15*, and *OsMADS18* (also known as *RAP1B*, *RAP1A*, and *OsMADS28*, respectively), which are classified as a monocot-specific lineage of *AP1*-like genes[13,14,15,16] (Fig. 1). Of these, *OsMADS14* and *OsMADS15* are classified as a pair of sister subclasses, and *OsMADS18* is classified as a relatively distant subclass of the monocot *AP1*-like gene family[17]. It will be interesting to determine whether these genes have functions similar to those of the *AP1*-like genes in eudicots because the rice floret does not have obvious sepals and petals, but instead has a lemma, a palea, and lodicules.

*OsMADS14* and *OsMADS15* are initially expressed across the whole region of the floral meristem in flower development. Subsequently, the expression domains of these genes are restricted to the primordia of glumes, lemma, palea, and lodicules[15,18]. Thus, the expression patterns of *OsMADS14* and *OsMADS15* in rice are similar to that of *AP1* in *Arabidopsis*. Ectopic expression of *OsMADS14* induces flower development from regenerated shoots at the early stages of development and, in an extreme case, directly from cells in the callus without vegetative growth[14]. This observation suggests that *OsMADS14* is involved in promoting flowering and determining the identity of the floral meristem in rice. Studies on loss-of-function or gain-of-function mutants of *OsMADS15* have not been reported so far.

The functions of *OsMADS18* may differ from those of *OsMADS14* and *OsMADS15* because the expression pattern of *OsMADS18* is different from those of *OsMADS14* and *OsMADS15*. *OsMADS18* is expressed in roots, leaves, inflorescences, and flowers, and its expression levels reach a maximum when the plant reaches the reproductive stage[19,20]. *OsMADS18* is expressed in all regions of the flower[18] and its overexpression induces early flowering with accelerated development of the axillary shoot meristem[19].

As a whole, the function of *AP1*-like MADS-box genes in rice is still unclear, as compared to other classes of MADS-box genes, probably owing to the lack of loss-of-function analysis and to the genetic redundancy of these genes.

## B-CLASS GENES IN RICE

In *Arabidopsis*, two B-class MADS-box genes, *APETALA3* (*AP3*) and *PISTILLATA* (*PI*), are required to specify petal and stamen identity, as are their respective orthologs *DEF* and *GLO* in *Antirrhinum majus*[21]. Genetic and molecular studies have revealed that the functions of the B-class genes are almost conserved in rice[22]. In the rice *superwoman1* (*spw1*) mutant, lodicules and stamens are homeotically transformed into palea-like organs and carpels, respectively, a phenotype similar to that of loss-of-function mutants of B-class genes in *Arabidopsis* or *Antirrhinum* (Fig. 3). Molecular cloning has revealed that *SPW1* is a B-class MADS-box gene (also known as *OsMADS16*), which is a rice ortholog of *AP3* and *DEF* (Fig. 1). In addition, *SWP1* is expressed in lodicule and stamen primordia from their initiation to the later stages of their development. Taken together, these observations indicate that the functions of *SPW1* and *AP3* are well conserved between rice and *Arabidopsis* (Fig. 4).

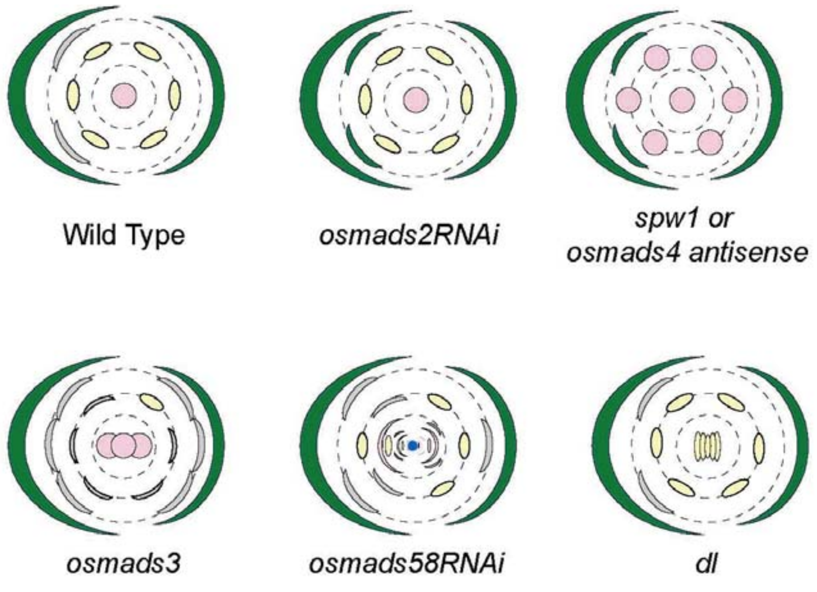

**FIGURE 3.** Phenotypes of the wild-type flower and floral homeotic mutants. Lemma, palea, and ectopic glume-like organs are indicated in green; lodicules, stamens and carpels are indicated in gray, yellow, and pink, respectively. The blue circle in the *osmads58RANi* flower indicates an indeterminate floral meristem, which produces reiterative sets of lodicules, stamens, and abnormal carpel-like organs.

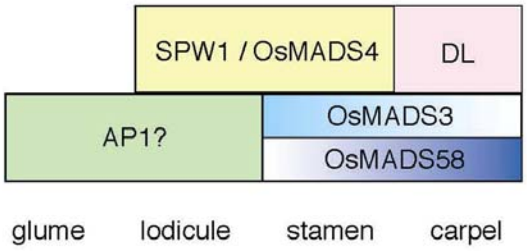

**FIGURE 4.** Current model of floral organ specification in rice.

The rice genome contains two *PI/GLO* orthologs, *OsMADS2* and *OsMADS4*, in contrast to its single *AP3* ortholog[23] (Fig. 1). *OsMADS4* is expressed in stamen and lodicule primordia. Antisense suppression of *OsMADS4* results in homeotic transformation of both lodicules and stamens into palea/lemma-like organs and carpels, respectively (Fig. 3), indicating that *OsMADS4* has a function similar to that of *PI/GLO* (Fig. 4).

The molecular relationship between *SPW1* and *OsMADS4* seems to be also conserved in rice. SPW1 and OsMADS4 proteins physically interact with each other in yeast two-hybrid analysis[24]. In addition, the expression of *OsMADS4* is upregulated in transgenic plants that ectopically express *SPW1*, and carpels are homeotically transformed into stamens in these transgenic plants[25]. These studies suggest that SPW1 and OsMADS4 work as a heterodimer and autoregulate their expression, similar to AP3 and PI in *Arabidopsis*[26]. Functional conservation of the B-class MADS-box genes in maize has been also demonstrated by genetic and biochemical studies[27,28]. Thus, it can be concluded that B-class MADS-box gene function is highly conserved among the angiosperms.

It seems that the function of *OsMADS2* slightly differs from that of *OsMADS4* in rice. *OsMADS2* is expressed in both lodicules and stamens, but its expression is stronger in lodicules than in stamens[15]. Because RNAi suppression of *OsMADS2* results in homeotic conversion of lodicules into palea/lemma-like organs, but stamens develop normally[29] (Fig. 3), it is possible that the function of *OsMADS2* has become more specialized to specify lodicule identity. OsMADS2 does not interact with SPW1 in yeast and OsMADS2 is not upregulated in transgenic plants that ectopically express *SPW1*[25]. Thus, the function of OsMADS2 protein also differs from that of OsMADS4 to a degree. Because duplication of genes in the *PI/GLO* family is common within the grass family[30], it will be interesting to determine whether this functional diversification of *PI/GLO* family genes has occurred in other grass species as well as in rice.

## C-CLASS GENES IN RICE

C-class genes, such as *Arabidopsis AGAMOUS* (*AG*) and *Antirrhinum PLENA* (*PLE*), play important roles in the specification of stamen and carpel identity, the control of floral meristem determinacy, and the negative regulation of A-function gene activity[21]. The rice genome contains two C-class genes, *OsMADS3* and *OsMADS58*, that arose through gene duplication[31,32] (Fig. 1). Molecular genetic studies on these two rice C-class genes have revealed further functional diversification of duplicated MADS-box genes[32]. *OsMADS3* and *OsMADS58* are expressed in the stamen and carpel whorl, like typical eudicot C-class genes, but their temporal patterns of expression differ from each other. *OsMADS3* is expressed only in the presumptive region of stamen and carpel primordia just before the initiation of these organs, whereas *OsMADS58* is expressed in the stamen and carpel whorls before initiation and during the development of stamen and carpel primordia. In an insertional knockout mutant of *OsMADS3*, stamens are homeotically transformed into lodicules, whereas carpels develop almost normally (Fig. 3). Thus, *OsMADS3* plays a predominant role in stamen specification (Fig. 4). By contrast, RNA-silenced lines of *OsMADS58* develop indeterminate flowers that reiterate a set of floral organs including lodicules, stamens, and abnormal carpel-like organs (Fig. 3). In these reiterated flowers, stamens develop almost normally, but carpels are morphologically abnormal. Thus, *OsMADS58* has a critical function both in the establishment of floral meristem determinacy and in normal carpel development (Fig. 4). These results indicate that the original functions of C-class genes have been partitioned into two paralogous genes, *OsMADS3* and *OsMADS58*, during the evolution of rice.

The mechanism underlying this functional diversification of C-class genes in rice may be partially explained by variation in the temporal expression of the two C-class genes. For example, *OsMADS3* is down-regulated in the floral meristem before meristem activity is terminated, whereas expression of *OsMADS58* is maintained even after carpel initiation. The predominant function of *OsMADS58* in floral meristem determinacy cannot be explained by its expression profile alone, however, because *OsMADS58* is expressed uniformly in both whorl 3 and whorl 4. Another possibility is that differences in the proteins that interact with the two MADS-domain proteins may have led to this functional diversification. That is, OsMADS3 and OsMADS58 may interact specifically and independently with different factors that are preferentially expressed in whorl 3 and whorl 4, respectively. The exact molecular mechanism underlying the functional diversification of *OsMADS3* and *OsMADS58* during the evolution of rice remains an interesting subject for future study.

Another role of the C-class genes in rice, revealed by functional studies, is the control of lodicule positioning[32]. In both a loss-of-function line of *OsMADS3* and an RNA-silencing line of *OsMADS58*, ectopic lodicules form at the palea side of whorl 2, leading to a radially symmetric arrangement of lodicules, whereas lodicules develop only at the lemma side of whorl 2 in wild type (Fig. 3). The spatial expression patterns of *OsMADS3* and *OsMADS58* are also asymmetric in the wild-type flower; that is, the two genes are down-regulated at the lemma side of whorl2 where lodicules develop, but are up-regulated in the region adjacent to the palea. These observations indicate that C-class genes in rice negatively regulate lodicule development at the palea side in the wild-type flower. Monocot flowers generally have a radially symmetric perianth and three lodicules arranged in radially symmetric positions is an ancestral phenotype in the grass family[33]. Therefore, it will be of great interest to reveal whether the asymmetric distribution of lodicules was correlated with the asymmetric expression of C-class genes during the evolution of grass flowers.

## CARPEL SPECIFICATION IN RICE

The genetic regulation of carpel specification in rice seems to differ from that in eudicots because carpels develop in loss-of-function lines of the C-class genes *OsMADS3* and *OsMADS58*, although they have an abnormal morphology in *osmads58* mutant lines. Carpel specification in rice largely depends, instead, on the activity of the gene *DROOPING LEAF* (*DL*)[34]. In loss-of-function mutants of *DL*, carpels are homeotically transformed into stamens (Fig. 3). *DL* encodes a *YABBY* transcription factor closely related to *Arabidopsis CRABS CLAW* (*CRC*) and expression of *DL* is specific to carpel primordia. Because the function of the *CRC/DL* subfamily of *YABBY* genes is conserved in angiosperms to a degree and regulates carpel development and morphogenesis[35,36,37], *DL* may have evolved a more essential role in carpel development, such as organ specification, during the evolution of rice (Fig. 4).

Recent studies have revealed genetic interactions between *DL* and MADS-box genes in rice flower development. First, expression studies using *dl* and *spw1* mutants indicate that *DL* and B-class genes negatively regulate each other[22,34]. Second, *DL* may function independently of C-class genes because abnormal carpel-like organs still develop in a double mutant derived from a weak loss-of-function mutant of *OsMADS3* and RNAi-suppression lines of *OsMADS58*. At present, however, the possibility cannot be ruled out that residual *OsMADS3* function in the double mutant induces the expression of *DL*. Generating a double mutant with severe loss of function of *OsMADS3* and *OsMADS58* and a triple mutant including a *dl* mutation would reveal the precise genetic interactions between *DL* and the C-class genes in rice.

## D-CLASS GENES IN RICE

*Arabidopsis SEEDSTICK* (*STK*) and its petunia orthologs, *FBP7* and *FBP11*, regulate ovule identity and these MADS-box genes are known as D-class genes[38,39]. In *Arabidopsis*, the D-class gene *STK* and the C-class genes *AG*, *SHP1*, and *SHP2* redundantly regulate the specification of ovule identity[39].

Studying D-class function and revealing how D-class genes are regulated in rice may provide clues to the wide variation in the number and position of ovules in the gynoecium observed among angiosperms. The rice genome contains two MADS-box genes, *OsMADS13* and *OsMADS21*, that show high similarity to D-class MADS-box genes in eudicots[2,40,41] (Fig. 1). *OsMADS13* is expressed specifically in ovule primordia and in the inner cell layer of the carpel wall[40]. In addition, OsMADS13 of rice and FBP7 of petunia interact with the same set of MADS-domain proteins in yeast[42]. These observations therefore suggest that the function of *OsMADS13* is similar to that of *FBP7* and that *OsMADS13* is probably involved in ovule specification in rice. Overexpression of *OsMADS13* does not result in the development of ectopic ovules in rice, however, unlike overexpression of the *FBP* genes in petunia[42]. Thus, *OsMADS13* alone may be insufficient to specify ovule identity in the rice flower, and the mechanism of ovule formation may differ slightly between rice and petunia.

The function and expression pattern of *OsMADS21* are currently unknown. Because the gene duplication event that produced *OsMADS13* and *OsMADS21* occurred before the divergence of rice and maize[41], it is possible that the functions of these genes may have diversified during evolution of the grass species.

## *SEP*-LIKE GENES IN RICE

*Arabidopsis SEPALLATA* genes (*SEP1*, *SEP2*, *SEP3*, and *SEP4*) form an integral part of the mechanisms underlying floral organ specification[43,44,45,46,47]. SEP proteins function as cofactors that provide flower-specific activity to *ABCD* genes by forming complexes with their products.

The *SEP*-like genes in the angiosperms are divided into two major clades: the *SEP3* clade and the *LOFSEP* clade[48]. The rice genome contains at least five *SEP*-like MADS-box genes: two of these, *OsMADS7* (also known as *OsMADS45*) and *OsMADS8* (*OsMADS24*), are classified as members of the *SEP3* clade, whereas the other three, *LHS1* (*OsMADS1)*, *OsMADS5,* and *OsMADS34*, are classified as members of the *LOFSEP* clade[2,48] (Fig. 1).

The only *SEP*-like gene whose function has been well characterized is *LHS1*[49,50,51,52,53]. This gene is first expressed in the floral meristem, but not in the empty glumes. Subsequently, *LHS1* is expressed strongly in the palea and lemma, and weakly in the carpel[50]. In the *lhs1* mutant or in RNAi suppression lines of *LHS1*, the lemma and palea are transformed into leaf-like organs, showing loss of identity of the palea and lemma[49,51,52,53]. Conversely, ectopic expression of *LHS1* causes homeotic transformation of the empty glumes into palea/lemma-like organs, together with other abnormalities, such as short panicles and irregularly positioned branches[49,50]. These results suggest that *LHS1* regulates palea/lemma identity in rice. Because the LHS1 protein physically interacts with the AP1-like MADS-domain proteins OsMADS14 and OsMADS15[16], its function may involve formation of a multiprotein complex with proteins encoded by A-class genes.

Another important function of *LHS1* is the regulation of meristem determinacy. Loss of function of *LHS1* causes a pleiotropic spikelet phenotype, which is probably caused by the loss of meristem determinacy[49,51,52,53]. In weak loss-of-function alleles, a pair of florets is formed in a spikelet, whereas in severe alleles, glume-like organs develop reiteratively. It has not been fully clarified in the literature[49,51,52,53] whether this abnormality is caused by defects in floral meristem determinacy or by defects in spikelet meristem determinacy or both, perhaps because it is difficult to distinguish between the two possibilities as the spikelet meristem is quickly converted into the floral meristem in rice. A few phenotypes, however, suggest that the defects are in determinacy of the spikelet meristem: first, the glume-like organs that are reiterated in the *lhs* mutant could be regarded as reduced florets; and second, pedicels sometimes develop in the *lhs1* floret[52]. Isolation of an *lhs1* mutant in species with multiple florets may aid our understanding of the function of *LHS*-like genes. Notably, in the grass family the expression of *LHS1* homologs varies from species to species with the developmental pattern of florets in the spikelet[54]. In several species with spikelets in which the florets develop basipetally (from top to bottom), *LHS1* homologs are expressed only in the terminal floret of the spikelet. In other species with spikelets in which the florets develop acropetally (from bottom to top), by contrast, *LHS1* homologs are expressed in multiple florets. Thus, these differences in *LHS1* expression may be associated with the developmental patterns of the spikelet, suggesting that *LHS1*-like genes may have been involved in morphological diversification of inflorescences during the evolution of grass species.

The functions of other *SEP*-like genes in rice are not fully established, partly because of the functional redundancy of these genes. For example, a loss-of-function mutant of *OsMADS5* does not show any obvious phenotype[52]. It has been suggested, however, that the functions of *SEP*-like genes in rice may have diversified to some degree. That is, the expression patterns of *SEP*-like genes in the rice flower are variable and the interactions of SEP-like proteins with the products of ABCD MADS-box genes are not identical[48]. Establishing the functional diversification of *SEP*-like genes in rice and deducing the roles of these genes in the morphological divergence of the floret, spikelet, and inflorescence would throw light on the evolution of the grass family.

## FUTURE PROSPECTS

Although several functional studies have identified the developmental roles of some MADS-box genes in rice, much remains to be studied. Isolation of loss-of-function mutants, and combinations of such mutants, will reveal the fine details of MADS-box gene function during rice development. In addition, analysis of the interactions of MADS-domain proteins with other types of protein including different MADS-domain proteins, especially *in planta*, will help to elucidate the molecular roles of MADS-box genes in rice. The mechanism underlying the functional diversification of duplicated MADS-box genes, such as *OsMADS3* and *OsMADS58*, and *OsMADS4* and *OsMADS2*, is also an interesting issue to be resolved. Such studies, along with similar analyses in other grass species, will further our understanding of the evolution and the diversification of grass flowers and inflorescences.

## BIOSKETCHES

**Takahiro Yamaguchi** is a Postdoctoral Fellow at the National Institute for Basic Biology, Myodaiji-cho Okazaki, Aichi 444-8585, Japan. Research interests include molecular genetic studies about flower and leaf development in *Oryza sativa* (rice) and *Arabidopsis thaliana*, EvoDevo studies about evolution of leaf form in monocots, and EcoDevo studies about leaf development in Arabidopsis using natural variations. E-mail: tyama@nibb.ac.jp

**Hiroyuki Hirano** is a Professor in the Graduate School of Science, University of Tokyo, Hongo, Bunkyo-ku, Tokyo 113-8654. Research interests include flower and leaf development in rice and monocots, molecular mechanism of meristem maintenance, evolution of flower morphology and its genetic regulation, and mobile elements and genome evolution. E-mail: hyhirano@biol.s.u-tokyo.ac.jp
